# Combined Effects of Smoking and Bilirubin Levels on the Risk of Lung Cancer in Korea: The Severance Cohort Study

**DOI:** 10.1371/journal.pone.0103972

**Published:** 2014-08-06

**Authors:** Jung-eun Lim, Heejin Kimm, Sun Ha Jee

**Affiliations:** 1 Institute for Health Promotion & Department of Epidemiology and Health Promotion, Graduate school of Public Health, Yonsei University, Seoul, Republic of Korea; 2 Department of public health, Graduate School, Yonsei University, Seoul, Republic of Korea; Kagoshima University Graduate School of Medical and Dental Sciences, Japan

## Abstract

**Background:**

Smoking is a major risk factor for lung cancer. Bilirubin, an antioxidant, is inversely associated with the risk of diseases related to oxidative stress. This study was conducted to determine the influence of smoking and bilirubin levels on the risk of lung cancer in the Severance cohort study.

**Methods:**

This study included 68,676 Korean who received a health examination at Severance Health Promotion Center from 1994 to 2004. Serum bilirubin measurements within normal range were divided into tertiles whereas smoking states were divided as never-smokers, former smokers and current smokers. A diagnosis of lung cancer was coded as occurring based on the report from the National Cancer Registry. Hazard ratios (HRs) and 95% confidence intervals (95% CIs) were calculated using Cox proportional hazards model.

**Results:**

At the end of the study period, 240 patients (men: 181, women: 59) developed lung cancer. Compared to those with bilirubin levels ≥1.0 mg/dL, HRs (95% CI) for lung cancer were 2.8 (1.8–4.2) for subjects having bilirubin levels from 0.2 to 0.7 mg/dL in men. When we stratified our analysis by smoking status, bilirubin consistently showed a protective effect on the risk of lung cancer on both never- and current smokers. Current smokers having bilirubin levels from 0.2 to 0.7 mg/dL had a risk of lung cancer by 6.0-fold higher than never-smokers with bilirubin levels ≥1.0 mg/dL in men.

**Conclusion:**

In this large prospective study, higher baseline bilirubin level in the normal range was associated with low risk of lung cancer. Smoking and low bilirubin levels were cumulatively associated with a higher risk of lung cancer.

## Introduction

Lung cancer is the most frequent cause of cancer death among men worldwide with an estimated age-adjusted mortality rate of 23.0 per 100,000 in the year 2008 [1]. In Korea, the mortality rate from lung cancer was 38.7 per 100,000 among men [2]. Lung cancer surpassed stomach cancer as the leading cause of cancer death in 1999 [2].

Cigarette smoking is the main epidemiologically proven cause of lung cancer [3], and approximate 80–90% of lung cancers are attributed to cigarette smoking [4]. Smoking is a preventable risk factor for disease burden worldwide and resulted in 5.1 million years of potential life lost annually in the United Stated during 2000–2004 [5].

Serum bilirubin, a bile pigment, is a major breakdown product of heme catabolism and thus highly related with hemoglobin [6], [7]. Bilirubin protects lipid membranes, protein albumin and other proteins, especially in areas of poor antioxidant defense systems such as the myocardium and nervous system [6]. From cross-sectional and longitudinal studies, serum bilirubin has inverse associations with the risk of cardiovascular disease (CVD), stroke, metabolic syndrome and cancers such as colorectal cancer and breast cancer [8]–[12]. An experimental study using animal models support a protective effect of increased bilirubin against respiratory injury by environmental stressors [13]. Given the remarkable antioxidant, cytoprotective, and antiinflammatory properties of bilirubin, and the critical role of oxidative stress, we hypothesized that higher baseline bilirubin level is associated with lower risk of lung cancer. Only one cohort study which used UK primary care data showed an inverse association between bilirubin and lung cancer, but we could not find a similar Asian population-based cohort study [14].

Smoking was associated with decreased serum bilirubin concentrations [15]. In the relationship between serum bilirubin and CVD, smoking was a strong risk factor that increased this association [16]. However, there were a few population based studies evaluating whether smoking may affect the association between serum bilirubin and cancers, particularly lung cancer.

This study was conducted to evaluate the relationship between serum bilirubin levels and the risk of lung cancer and to examine the combined effects of serum bilirubin and smoking on the risk of lung cancer among Korean adults (age≥20 years) in The Severance cohort study.

## Materials and Methods

### Study subjects

The Severance cohort study included 78,615 participants (men: 40,142, women: 38,473) aged 20–93 years Korean who visited the Health Promotion Centers at Severance Hospital located in Seoul for routine examinations from 1994 to 2004 [17]–[21]. This prospective cohort study started follow-up when people are enrolled during baseline (1994–2004) and followed up cancer events until 2009. People who already have cancer (n = 868), or past history of cancer (n = 53) were excluded. We additionally excluded subjects (N = 609) who got lung cancer before or up to 3 years after the date of the bilirubin measurement. Subjects with missing data in the major variables (n = 5,195), hemoglobin level<10 g/dL or >20 g/dL (n = 831), and patients with bilirubin values below 0.18 mg/dL (3 µmol/L) for both sexes were excluded (n = 9) [14]. Among the 71,050 participants, the potential Gilbert syndrome group (total bilirubin >34.2 umol/L [2.0 mg/dL], aspartate aminotransferase <80 IU/L, alanine transaminase <80 IU/L, and gamma glutamyl transpeptidase [GGT] <80 IU/L; n = 665) and potential hepatobiliary disease group (total bilirubin >34.2 umol/L or aspartate aminotransferase ≥80 IU/L or alanine aminotransferase ≥80 IU/L or serum albumin <3.5 g/dL; n = 1,709) were also excluded to avoid confounding factors. The potential Gilbert syndrome group and the potential hepatobiliary disease group were defined according to the previous studies [9], [22]. Therefore, a total of 68,676 participants were included in this study (Figure S1). The Institutional Review Board of Human Research of Yonsei University approved this study.

### Data collection

Demographic characteristics including age, gender, family history and past history of clinical diseases, as well as cigarette smoking status (never-smoker, former smoker or current smoker) and alcohol consumption status (never-drinker and ever-drinker) were collected via a standardized health questionnaire during a standardized examination at Severance Hospital. Both current- and former-smokers were asked to report the average number of cigarettes per day they smoked at the time or had smoked earlier. The body mass index (BMI) was calculated as weight divided by the square of height (m^2^).

For clinical chemistry assays, serum was separated from peripheral venous blood samples obtained from each participant after 12 hours of fasting. Serum bilirubin, fasting blood glucose, total cholesterol, and high-density lipoprotein cholesterol (HDL-C) were measured using a Hitachi-7600 analyzer (Hitachi Ltd., Tokyo, Japan).

All measurements were performed by a central laboratory at Severance Hospital, Yonsei University Health System, Seoul, Korea. Data quality control was maintained in accordance with the procedures of the Korean Association of Laboratory Quality Control.

### Outcome variables and follow-up

The principle outcome variable was incident lung cancer, based on National Cancer Registry. The Korean Ministry of Health and Welfare started a nationwide, hospital-based cancer registry in 1980 [23]. More than 180 hospitals are currently participating, and the data covers approximately 99% of new cancer cases in Korea. Cases were identified through a personal identification number and other usual identification variables such as names and addresses. The list of registered cases and a list of cancer cases from claims made through the National Health Insurance Corporation for each region were sent to the regional cancer registries to find any dropped cases [23]. Cancer cases were classified according to the International Classification of Diseases, 10th edition (ICD-10), and lung cancer was coded as C33-34 [23], [24]. We followed up lung cancer events from January 1995 to June 2009. Subjects diagnosed as cancers other than lung cancer during follow-up or any confirmed death within data provided by the National Statistical Office were treated as censored in this study. We were not able to take the issue of migration into account.

### Statistical analysis

Data were expressed as mean ± standard deviation (SD). Chi-square test and general linear models were used for analysis of statistical differences among the characteristics of the study participants. To confirm combined effects of smoking status (never-smoker, former smoker, and current smoker) and bilirubin levels on the risk of lung cancer, serum bilirubin concentrations were classified into tertiles (men: 0.2≤T1<0.8 mg/dL, 0.8≤T2<1.0 mg/dL, 1.0≤T3<2.9 mg/dL; women: 0.2≤T1<0.6 mg/dL 0.6≤T2<0.8 mg/dL, 0.8≤T3<2.9 mg/dL). Heavy smoker is defined by a daily cigarettes consumption of more than 20 pieces according to the recommendations of the World Health Organization (WHO).

To examine the association between smoking, bilirubin levels and the risk of lung cancer, Cox proportional hazard models were examined after adjusting for age and other potential confounding factors, including body mass index (BMI), white blood cell count, hemoglobin, and alcohol intake. Cox proportional hazard models were also used to calculate the risk of having lung cancer comparing the highest and lowest tertiles of bilirubin by smoking status. All analyses were performed using SAS statistical software, version 9.1 (SAS Institute Inc., Cary, NC). All statistical tests were two sided, and null hypotheses of no difference were rejected if p-values were less than .05, or, equivalently, if the 95% CIs of hazard ratio estimates excluded 1.

## Results

Recruited into the study were 68,676 patients from 78,615, with a median follow-up of 8.95 years. At the end of the study period, 240 patients (men: 181, women: 59) developed lung cancer (Table 1). In the baseline characteristics of study subjects, after adjustment for age, there were significant differences in BMI, serum bilirubin, white blood cell count, HDL-C, smoking status, and alcohol consumption status between the men with and those without lung cancer (p-value<0.05) (Table 1). However, there were significant differences in age, white blood cell count, systolic blood pressure, HDL-C, and triglyceride between the same groups in women (p value <0.05).

**Table 1:** Baseline characteristics of the study population (N = 68,676), 1994–2004^a,b^.

	Men			Women		
	N = 35,467			N = 33,209		
	Lung cancer	Non-lung cancer		Lung cancer	Non-lung cancer	
	N = 181	N = 35,286	p value	N = 59	N = 33,150	p value
Age, year	57.6 (0.8)	45.3 (0.1)	<0.0001	55.8 (1.6)	45.7 (0.1)	<0.0001
Body mass index, kg/m^2^	23.6 (0.2)	24.2 (0.02)	0.0162	23.1 (0.4)	23.2 (0.02)	0.6521
Serum bilirubin, mg/dl	0.8 (0.02)	0.9 (0.002)	<0.0001	0.7 (0.04)	0.7 (0.002)	0.5548
Hemoglobin, g/dl	15.3 (0.1)	15.2 (0.01)	0.3591	13.0 (0.1)	13.0 (0.01)	0.4513
White blood cell count, ×10^3^/ul	7.5 (0.1)	6.9 (0.01)	<0.0001	6.6 (0.2)	6.1 (0.01)	0.0166
Aspartate aminotransferase, IU/L	20.6 (0.6)	21.5 (0.04)	0.1273	18.5 (0.9)	18.6 (0.04)	0.9606
Alanine aminotransferase, IU/L	24.2 (1.04)	25.6 (0.1)	0.1780	16.2 (1.2)	16.8 (0.1)	0.6274
Gamma-glutamyl transpeptidase, IU/L	45.0 (3.3)	40.7 (0.2)	0.1949	15.7 (2.2)	17.9 (0.1)	0.3212
Systolic blood pressure, mmHg	126.0 (1.3)	123.7 (0.1)	0.7060	125.0 (2.3)	119.8 (0.1)	0.0240
Diastolic blood pressure, mmHg	72.2 (0.9)	73.3 (0.1)	0.2014	72.4 (1.5)	72.2 (0.1)	0.8734
Total cholesterol, mg/dl	194.1 (2.6)	194.9 (0.2)	0.7802	193.9 (4.4)	193.3 (0.2)	0.8796
HDL cholesterol, mg/dl	45.0 (0.9)	47.1 (0.1)	0.0123	50.6 (1.7)	54.6 (0.1)	0.0180
Triglyceride, mg/dl	164.9 (8.2)	163.2 (0.6)	0.8384	152.0 (9.7)	119.7 (0.4)	0.0008
Fasting blood sugar, mg/dl	98.6 (1.8)	97.4 (0.1)	0.5144	91.5 (2.6)	92.7 (0.1)	0.6333
Condition, %						
Smoking status						
Ex	26.5	28.5		6.8	2.9	
Current	64.1	50.9	0.0002	10.2	6.3	0.0824
Alcohol drinking (yes)	74.0	83.4	0.0008	25.4	32.2	0.2667
Family history of cancer (yes)	27.1	24.2	0.3648	25.4	25.1	0.9506

a Data are expressed as mean (standard error) unless otherwise indicated.

b Except for age, all mean values were age-adjusted.

Abbreviation: HDL, high-density lipoprotein.

Table 2 shows associations of various variables with bilirubin tertiles at baseline among men and women. After adjusting for age, there were consistent negative relationships of bilirubin tertiles with body mass index, white blood cell count, AST, ALT, triglyceride and current smoking in both sexes. However, HDL-C, hemoglobin, and alcohol drinking status were positively related to bilirubin levels.

**Table 2:** Association of various variables according to serum bilirubin levels, 1994–2004^a^.

	Serum bilirubin in men				Serum bilirubin in women			
	T3	T2	T1		T3	T2	T1	
	Mean	Mean	Mean	P for trend	Mean	Mean	Mean	P for trend
Body mass index, kg/m^2^	24.0	24.3	24.2	<0.0001	23.1	23.2	23.4	<0.0001
White blood cell count, ×10^3^/ul	6.6	6.9	7.1	<0.0001	6.1	6.1	6.2	<0.0001
Aspartate aminotransferase, IU/L	21.3	21.5	21.6	<0.0001	18.4	18.4	19.0	<0.0001
Alanine aminotransferase, IU/L	24.9	26.0	26.0	<0.0001	16.7	16.6	17.2	<0.0001
HDL cholesterol,	47.9	47.2	46.3	<0.0001	55.3	54.5	54.0	<0.0001
Hemoglobin	15.4	15.3	15.0	<0.0001	13.1	13.0	12.8	<0.0001
Triglyceride	154.9	165.2	169.8	<0.0001	113.2	117.9	127.6	<0.0001

	%	%	%		%	%	%	
Smoking status								
Ex	30.3	29.3	26.3		2.5	3.1	3.0	
Current	46.2	50.2	56.1	<0.0001	5.3	5.9	7.5	<0.0001
Alcohol drinking (yes)	85.4	84.3	80.8	<0.0001	34.1	32.0	30.6	<0.0001
Family history of cancer (yes)	24.7	24.3	23.7	0.2038	24.7	25.1	25.4	0.5129

a All mean values were age-adjusted.

Abbreviation: HDL, high-density lipoprotein.

T3: highest tertile, T2: middle tertile, T1: lowest tertile.

In multivariate Cox proportional hazard models, after adjusting for age, BMI, white blood cell count, hemoglobin, and alcohol intake, the lowest bilirubin tertile was significantly associated with the risk of lung cancer compared to those in the highest tertile for men [HR(95% CI) = 2.8 (1.8–4.2)] (Table 3). After additional adjustment for smoking status, there was no appreciable change found in the risk [HR(95% CI) = 2.5 (1.7–3.7)]. Current smoking was as expected positively significantly associated with the risk of lung cancer in both men and women (Table S1). In heavy smokers, HR was increased up to 4.0 compared to never-smokers among men (Table S2).

**Table 3:** HR and 95% CI for lung cancer according to serum bilirubin levels^a^.

	Men					Women				
	PY	Lung cancer	Rate per 10,000	HR (95% CI)^b^	HR (95% CI)	PY	Lung cancer	Rate per 10,000	HR (95% CI)^b^	HR (95% CI)
**Bilirubin level (mg/dl)**										
T3	122109.6	32	2.6	1.0	1.0	101483.9	19	1.9	1.0	1.0
T2	81282.7	45	5.5	1.8 (1.2–2.9)	1.9 (1.2–3.0)	96530.8	18	1.9	0.9 (0.5–1.7)	0.9 (0.5–1.7)
T1	116718.1	104	8.9	2.5 (1.7–3.7)	2.8 (1.8–4.2)	98304.0	22	2.2	1.0 (0.5–1.9)	1.0 (0.5–1.9)
P for trend				0.0007	<0.0001				0.5006	0.5964
1SD increase				0.7 (0.5–0.8)	0.6 (0.5–0.8)				1.0 (0.8–1.3)	1.0 (0.8–1.3)
1 mg/dl increase				0.3 (0.2–0.5)	0.2 (0.1–0.4)				1.0 (0.4–2.7)	1.0 (0.4–2.6)

a Adjusted for age, body mass index, white blood cell count, hemoglobin, and alcohol intake.

b Additionally adjusted for smoking status.

Abbreviation: PY, person year; SD, standard deviation; T3, highest tertile; T2, middle tertile; T1, lowest tertile.

In table 4, we observed the association between serum bilirubin level and the risk of lung cancer stratified by smoking status in men. Regardless of smoking status, we observed similar strength of negative associations between bilirubin and lung cancer. After we additionally adjusted for the amount of smoking, bilirubin consistently showed significant inverse association on the risk of lung cancer among current smokers. When we divided current smokers into light/medium smokers(<20cig/day) and heavy smoker(≥20cig/day), we could confirm the stronger significant association between serum bilirubin levels(per 1SD) and lung cancer risk in heavy smokers compared to light/medium smokers (light/medium smoker, HR (95%CI) = 0.70 (0.47–1.03); heavy smoker, HR (95%CI) = 0.67 (0.52–0.87)) (Data not shown).

**Table 4:** The association between serum total bilirubin level and the risk of lung cancer according to smoking status among Korean men^a^.

	HR (95% CI)
**Never-smoker**	
Number of total/case	7278/17
1SD increase	0.6 (0.3–1.0)
**Former smoker**	
Number of total/case	10108/48
1SD increase	0.7 (0.5–1.0)
**Current smoker** b	
Number of total/case	18081/116
1SD increase	0.7 (0.5–0.9)

a Adjusted for age, body mass index, white blood cell count, hemoglobin, and alcohol intake.

b Additionally adjusted for amount of smoking(per sig/day). 706 were excluded because of missing variable on amount of smoking.

**Figure 1:**
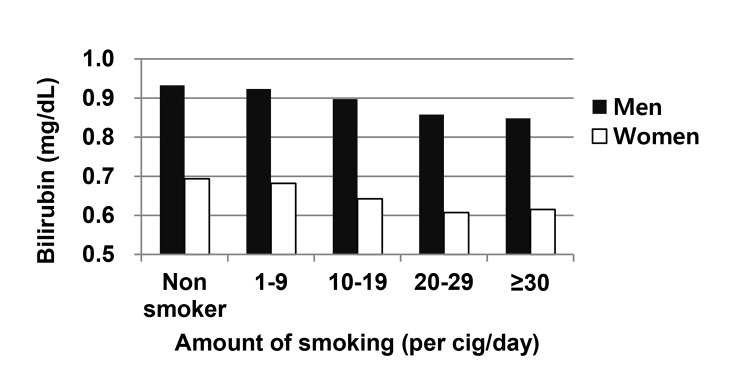
Serum bilirubin levels according to the amount of smoking.

In the distribution of bilirubin levels by the amount of smoking (per cig/day), concentrations of serum bilirubin decreased with increasing numbers of cigarettes smoked per day in both men and women (Figure 1).

**Figure 2:**
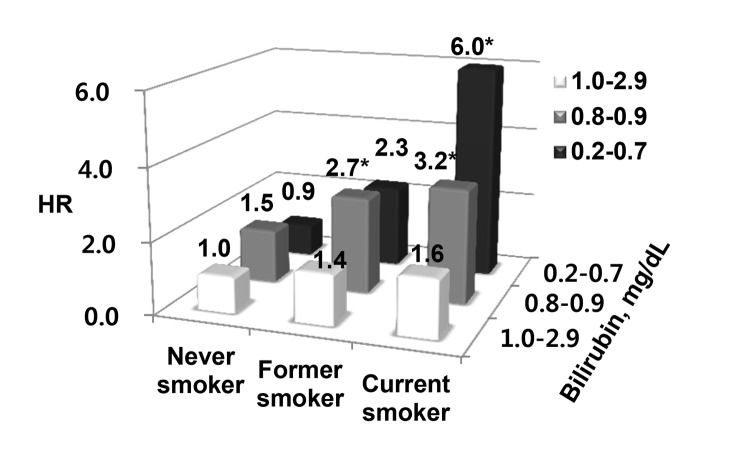
Hazard ratio(HR) for lung cancer according to serum bilirubin levels and smoking status in men. Cox proportional hazard models were examined after adjusting for age, body mass index, white blood cell count, hemoglobin, and alcohol intake. *represents 95% confidence interval of hazard ratio estimate excluded 1.

Figure 2 illustrates the combined effects of serum billirubin and smoking status with the risk of lung cancer in men. We found additive model when we examining the modifying effect of smoking on the association between serum bilirubin levels and lung cancer risk. After adjusting for the confounders, HR(95% CI) for the risk of lung cancer among current smokers was 1.6 (0.6–4.3). This HR increased to 6.0 when current smokers had the lowest tertile of bilirubin. Current smokers with the lowest tertile of bilirubin had a 3.8-fold increase in the risk of lung cancer. It was about 6.7-fold increase in the risk of lung cancer among participants with the lowest tertile of bilirubin. The interactions of the association between bilirubin and lung cancer with smoking status were not significant. When we carried out a sub-analysis restricted to men older than 50 years of age (N = 12,524), The HR increased to 7.7 when current smokers had the lowest tertile of bilirubin (Figure S2).

## Discussion

After accounting for the other important health indicators including smoking status, there was an inverse association between serum bilirubin levels and the incidences of lung cancer in men. Furthermore, when we combined the effect of smoking and bilirubin, the risk of lung cancer of current smokers with the lowest bilirubin tertile, compared to never smoker with the highest bilirubin tertile was significantly increased. To our knowledge, this is the first analysis of the combined effect of smoking and bilirubin on the risk of lung cancer.

While bilirubin has been generally regarded as little more than a metabolic by-product of heme degradation, epidemiologic studies suggest a protective effect of bilirubin against cancer and cancer-related death. One of the studies showed that each 1-mg/dL increase in serum bilirubin is associated with a markedly decreased prevalence of colorectal cancer (OR = 0.257; 95% CI 0.254–0.260) [25]. However, we can find only few previous studies about the association between bilirubin and lung cancer. A study showed an inverse association between bilirubin levels and the incidences of respiratory disease including lung cancer, which is consistent with our study [14]. In that study, regression estimates for incidence rate of lung cancer per 0.1-mg/dL increase in bilirubin level were an 8% decrease for men and an 11% decrease for women [14]. In our study, each 0.1 mg/dL increase in bilirubin level was significantly associated with 8% decrease in the risk of lung cancer incidence for men, and this study lacks statistical power to demonstrate associations in women. Unlike most previous studies, we provided Asian based information about the association between bilirubin and lung cancer. Moreover, we also evaluated whether smoking status may affect the association. Among never-smokers, serum bilirubin was consistently inversely associated with the risk of lung cancer. Among current smokers, after we additionally adjusted for the amount of smoking, bilirubin consistently showed significant protective effect on the risk of lung cancer.

We observed increased HR of 3.8 for lung cancer in current smoker compared with never-smoker in men (Table S1). Compared with the HRs between smoking and lung cancer from non-Asian based studies, this study showed small effect of smoking on lung cancer. However, it is consistent with previous Asian based studies. A meta-analysis study which investigated the association between smoking and all lung cancer showed HRs ranging from 1.22 to 5.10 in Asia including Japan and China [26]. A previous 16-year follow-up Korean cohort study suggested HR (95%CI); 4.1 (2.1–7.9) in current smoker compared with never-smoker [27].

In previous studies, the association between smoking and serum bilirubin concentration has been described [28], [29]. Total serum bilirubin level was lower in male smokers and intermediate in former-smokers compared with never-smokers in a study among the Belgian population. However, in female smokers, lower concentration of total serum bilirubin was found but the difference was not significant [29].

Smoking is found to be positively associated with hemoglobin, which regulates bilirubin concentrations [30], [31]. Heme oxygenase (HO)-1, the enzyme catalyzes the degradation of heme groups into equimolar amounts of biliverdin, is considered an antioxidant and cytoprotective enzyme [32]. Therefore, differences in bilirubin level may be due to the concentration of hemoglobin. Decreased survival of lung cancer was observed in low hemoglobin levels in previous study. The group of patients with hemoglobin level lower than percentile 25 had a survival rate that was below 41% [33]. In our study, we excluded participants who had abnormal hemoglobin levels and we additionally adjusted for hemoglobin in analyses, so our results are independent of hemoglobin effect.

Decreased mitochondrial functions by bilirubin and apoptotic action of bilirubin were consistently observed in previous studies [6], [34]. Bilirubin is regarded as a pro-oxidant showing its cytopathic effect on TMK-1, and growth inhibition close to 50%. An arrest at G0/G1 was shown by the analysis of the cell cycle [34]. The genetic variations of UGT1A1 can also be associated with the effect of bilirubin on the diseases related to oxidative stress [35].

This study has the following limitations: (i) The representativeness of the background population is limited because study subjects were recruited from individuals who went to the health promotion center to check their health status. (ii) In this study, we were not able to take migration information into account. Because the migration rate in Korea is less than 1%, this may not significantly impact the result of this study. (iii) Bilirubin concentrations were measured only once. (iv) Analysis for combined effect of smoking and bilirubin on the risk of lung cancer was performed only among men due to the small number of female Korean smokers. However, our study has a few strengths. First, we analyzed a large population with men and women. Second, the characteristics of participants were homogenous because they were all urban middle class. Finally, we found the combined effect of smoking and bilirubin on the risk of lung cancer for the first time.

In conclusion, this prospective study showed higher baseline bilirubin level whitin the normal range was associated with low risk of lung cancer in Korean men. The highest risk of lung cancer was observed in participants with very low levels of serum bilirubin and current male smokers.

A bilirubin assay is available in many laboratories and is not expensive. The results in this large cohort study support the potential role of bilirubin to predict lung cancer. Our findings suggest that serum bilirubin might have some protective function against lung cancer. Furthermore, when it combined with smoking control, it might be more effective. Further studies are needed to confirm the association of bilirubin with various cancers including lung cancer. Intervention trials may answer the question of the potential for bilirubin as a therapeutic target for lung cancer.

## Supporting Information

Figure S1 **Study subjects.**
(TIF)

Figure S2 **Hazard ratio(HR) for lung cancer according to serum bilirubin levels and smoking status in men older than 50 years of age (N = 12,524).** Cox proportional hazard models were examined after adjusting for age, body mass index, white blood cell count, hemoglobin and alcohol intake. *represents 95% confidence interval of hazard ratio estimate excluded 1.
(TIF)

Table S1 **HR and 95% CI for lung cancer according to smoking status.**
(DOCX)

Table S2 **HR and 95% CI for lung cancer according to smoking status and smoking amounts.**
(DOCX)

## References

[1] Ferlay J, Shin HR, Bray F, Forman D, Mathers C, et al (2010) Estimates of worldwide burden of cancer in 2008: GLOBOCAN 2008. Int J Cancer 127: 2893–2917. 21351269 10.1002/ijc.25516

[2] Jung KW, Park S, Kong HJ, Won YJ, Lee JY, et al (2011) Cancer statistics in Korea: incidence, mortality, survival, and prevalence in 2008. Cancer Res Treat 43: 1–11. 21509157 10.4143/crt.2011.43.1.1 PMC3072529

[3] Stampfli MR, Anderson GP (2009) How cigarette smoke skews immune responses to promote infection, lung disease and cancer. Nat Rev Immunol 9: 377–384. 19330016 10.1038/nri2530

[4] Rodrigues M, Havlik E, Peskar B, Sinzinger H (1998) Prostaglandins as biochemical markers of radiation injury to the salivary glands after iodine-131 therapy? Eur J Nucl Med 25: 265–269. 9580860 10.1007/s002590050227

[5] Centers for Disease Control and Prevention (2009) State-specific smoking-attributable mortality and years of potential life lost–United States, 2000–2004. MMWRMorb Mortal Wkly Rep 58: 29–33. 19165137

[6] Stocker R, Yamamoto Y, McDonagh AF, Glazer AN, Ames BN (1987) Bilirubin is an antioxidant of possible physiological importance. Science 235: 1043–1046. 3029864 10.1126/science.3029864

[7] Huang SS, Huang PH, Wu TC, Chen JW, Lin SJ (2012) Association of serum bilirubin with contrast-induced nephropathy and future cardiovascular events in patients undergoing coronary intervention. PLoS One 7: e42594. 22880046 10.1371/journal.pone.0042594 PMC3412818

[8] Schwertner HA, Vítek L (2009) Gilbert syndrome, UGT1A1*28 allele, and cardiovascular disease risk: Possible protective effects and therapeutic applications of bilirubin. Atherosclerosis 198: 1–11. 10.1016/j.atherosclerosis.2008.01.001 18343383

[9] Kimm H, Yun JE, Jo J, Jee SH (2009) Low serum bilirubin level as an independent predictor of stroke incidence: a prospective study in Korean men and women. Stroke 40: 3422–3427. 19713538 10.1161/STROKEAHA.109.560649

[10] Lin LY, Kuo HK, Hwang JJ, Lai LP, Chiang FT, et al (2009) Serum bilirubin is inversely associated with insulin resistance and metabolic syndrome among children and adolescents. Atherosclerosis 203: 563–568. 18775539 10.1016/j.atherosclerosis.2008.07.021

[11] Keshavan P, Schwemberger SJ, Smith DL, Babcock GF, Zucker SD (2004) Unconjugated bilirubin induces apoptosis in colon cancer cells by triggering mitochondrial depolarization. Int J Cancer 112: 433–445. 15382069 10.1002/ijc.20418

[12] Chang Y, Ryu S, Zhang Y, Son HJ, Kim JY, et al (2012) A cohort study of serum bilirubin levels and incident non-alcoholic fatty liver disease in middle aged Korean workers. PLoS One 7: e37241. 22615952 10.1371/journal.pone.0037241 PMC3352875

[13] Ryter SW, Morse D, Choi AM (2007) Carbon monoxide and bilirubin: potential therapies for pulmonary/vascular injury and disease. Am J Respir Cell Mol Biol 36: 175–182. 16980550 10.1165/rcmb.2006-0333TR PMC2176112

[14] Horsfall LJ, Rait G, Walters K, Swallow DM, Pereira SP, et al (2011) Serum bilirubin and risk of respiratory disease and death. JAMA 305: 691–697. 21325185 10.1001/jama.2011.124

[15] Jo J, Kimm H, Yun JE, Lee KJ, Jee SH (2012) Cigarette smoking and serum bilirubin subtypes in healthy Korean men: the Korea Medical Institute study. J Prev Med Public Health 45: 105–112. 22509451 10.3961/jpmph.2012.45.2.105 PMC3324713

[16] Djousse L, Levy D, Cupples LA, Evans JC, D'Agostino RB, et al (2001) Total serum bilirubin and risk of cardiovascular disease in the Framingham offspring study. Am J Cardiol 87: 1196–1200. 11356398 10.1016/s0002-9149(01)01494-1

[17] Hallan SI, Matsushita K, Sang Y, Mahmoodi BK, Black C, et al (2012) Age and association of kidney measures with mortality and end-stage renal disease. JAMA 308: 2349–2360. 23111824 10.1001/jama.2012.16817 PMC3936348

[18] Fox CS, Matsushita K, Woodward M, Bilo HJ, Chalmers J, et al (2012) Associations of kidney disease measures with mortality and end-stage renal disease in individuals with and without diabetes: a meta-analysis. Lancet 380: 1662–1673. 23013602 10.1016/S0140-6736(12)61350-6 PMC3771350

[19] Mahmoodi BK, Matsushita K, Woodward M, Blankestijn PJ, Cirillo M, et al (2012) Associations of kidney disease measures with mortality and end-stage renal disease in individuals with and without hypertension: a meta-analysis. Lancet 380: 1649–1661. 23013600 10.1016/S0140-6736(12)61272-0 PMC3993095

[20] Mok Y, Won S, Kimm H, Nam C, Ohrr H, et al (2012) Physical activity level and risk of death: the severance cohort study. J Epidemiol 22: 494–500. 22850543 10.2188/jea.JE20110110 PMC3798560

[21] Mok Y, Lee SJ, Kim MS, Cui W, Moon YM, et al (2012) Serum uric acid and chronic kidney disease: the Severance cohort study. Nephrol Dial Transplant 27: 1831–1835. 21940488 10.1093/ndt/gfr530

[22] Inoguchi T, Sasaki S, Kobayashi K, Takayanagi R, Yamada T (2007) Relationship between Gilbert syndrome and prevalence of vascular complications in patients with diabetes. JAMA 298: 1398–1400. 17895455 10.1001/jama.298.12.1398-b

[23] Shin HR, Won YJ, Jung KW, Kong HJ, Yim SH, et al (2005) Nationwide cancer incidence in Korea, 1999∼2001; first result using the national cancer incidence database. Cancer Res Treat 37: 325–331. 19956367 10.4143/crt.2005.37.6.325 PMC2785938

[24] Riaz SP, Horton M, Kang J, Mak V, Luchtenborg M, et al (2011) Lung cancer incidence and survival in England: an analysis by socioeconomic deprivation and urbanization. JThorac Oncol 6: 2005–2010. 21892107 10.1097/JTO.0b013e31822b02db

[25] Zucker SD, Horn PS, Sherman KE (2004) Serum bilirubin levels in the U.S. population: gender effect and inverse correlation with colorectal cancer. Hepatology 40: 827–835. 15382174 10.1002/hep.20407

[26] Lee PN, Forey BA, Coombs KJ (2012) Systematic review with meta-analysis of the epidemiological evidence in the 1900s relating smoking to lung cancer. BMC Cancer 12: 385–390. 22943444 10.1186/1471-2407-12-385 PMC3505152

[27] Bae JM, Li ZM, Shin MH, Kim DH, Lee MS, et al (2013) Lung cancer incidence by smoking status in Korean men: 16-years of observations in the Seoul Male Cancer Cohort study. J Korean Med Sci 28: 636–637. 23580076 10.3346/jkms.2013.28.4.636 PMC3617322

[28] Schwertner HA (1998) Association of smoking and low serum bilirubin antioxidant concentrations. Atherosclerosis 136: 383–387. 9543110 10.1016/s0021-9150(97)00232-3

[29] Van Hoydonck PG, Temme EH, Schouten EG (2001) Serum bilirubin concentration in a Belgian population: the association with smoking status and type of cigarettes. Int J Epidemiol 30: 1465–1472. 11821365 10.1093/ije/30.6.1465

[30] Micozzi MS, Albanes D, Stevens RG (1989) Relation of body size and composition to clinical biochemical and hematologic indices in US men and women. Am J Clin Nutr 50: 1276–1281. 2596419 10.1093/ajcn/50.6.1276

[31] Milman N, Byg KE, Mulvad G, Pedersen HS, Bjerregaard P (2001) Haemoglobin concentrations appear to be lower in indigenous Greenlanders than in Danes: assessment of haemoglobin in 234 Greenlanders and in 2804 Danes. Eur J Haematol 67: 23–29. 11553263 10.1034/j.1600-0609.2001.067001023.x

[32] Castilho Á, Aveleira CA, Leal EC, Simões NF, Fernandes CR, et al (2012) Heme oxygenase-1 protects retinal endothelial cells against high glucose- and oxidative/nitrosative stress-induced toxicity. PLoS One 7: e42428. 22879979 10.1371/journal.pone.0042428 PMC3411771

[33] García Prim JM, González Barcala FJ, Moldes Rodríguez M, Alvarez Dobañob JM, Hervada Vidal X, et al (2008) [Impact of hemoglobin level on lung cancer survival]. Med Clin (Barc) 131: 601–604. 19080849 10.1157/13127916

[34] Rao P, Suzuki R, Mizobuchi S, Yamaguchi T, Sasaguri S (2006) Bilirubin exhibits a novel anti-cancer effect on human adenocarcinoma. Biochem Biophys Res Commun 342: 1279–1283. 16516158 10.1016/j.bbrc.2006.02.074

[35] Milton JN, Sebastiani P, Solovieff N, Hartley SW, Bhatnagar P, et al (2012) A genome-wide association study of total bilirubin and cholelithiasis risk in sickle cell anemia. PLoS One 7: e34741. 22558097 10.1371/journal.pone.0034741 PMC3338756

