# Supplementary figures and images for: Combined Effects of Smoking and Bilirubin Levels on the Risk of Lung Cancer in Korea: The Severance Cohort Study
Source: PLoS One. 2014 Aug 6;9(8):e103972. doi: 10.1371/journal.pone.0103972 (PMC4123988; doi:10.1371/journal.pone.0103972)

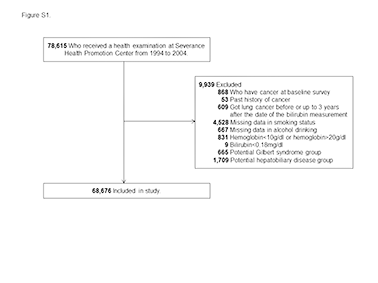

Supplement: Figure S1 — Study subjects. (TIF) [file pone.0103972.s001.tif]

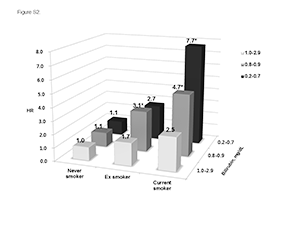

Supplement: Figure S2 — Hazard ratio(HR) for lung cancer according to serum bilirubin levels and smoking status in men older than 50 years of age (N = 12,524). Cox proportional hazard models were examined after adjusting for age, body mass index, white blood cell count, hemoglobin and alcohol intake. *represents 95% confidence interval of hazard ratio estimate excluded 1. (TIF) [file pone.0103972.s002.tif]
