# Supplementary material for: Combined Effects of Smoking and Bilirubin Levels on the Risk of Lung Cancer in Korea: The Severance Cohort Study
Source: PLoS One. 2014 Aug 6;9(8):e103972. doi: 10.1371/journal.pone.0103972 (PMC4123988; doi:10.1371/journal.pone.0103972)
Supplement: Table S1 — HR and 95% CI for lung cancer according to smoking status. (DOCX) [file pone.0103972.s003.docx]

Table S1. HR and 95% CI for lung cancer according to smoking status ^a^

|  | **Men** | | | | |  | **Women** | | | | |
| --- | --- | --- | --- | --- | --- | --- | --- | --- | --- | --- | --- |
|  | **PY** | **Lung** | **Rate per** | **HR (95% CI)** | **HR (95% CI)** ^b^ |  | **PY** | **Lung** | **Rate per** | **HR (95% CI)** | **HR (95% CI)** ^b^ |
|  |  | **cancer** | **10,000** |  |  |  |  | **cancer** | **10,000** |  |  |
|  |  |  |  |  |  |  |  |  |  |  |  |
| **Smoking** |  |  |  |  |  |  |  |  |  |  |  |
| **status** |  |  |  |  |  |  |  |  |  |  |  |
| Never-smoker | 65107.1 | 17 | 2.6 | 1.0 | 1.0 |  | 270000.3 | 49 | 1.8 | 1.0 | 1.0 |
| Former smoker | 88484.8 | 48 | 5.4 | 1.9 (1.1-3.3) | 1.9 (1.1-3.4) |  | 8031.3 | 4 | 5.0 | 2.9 (1.0-8.4) | 2.0 (0.8-4.9) |
| Current smoker | 166518.5 | 116 | 7.0 | 3.4 (2.0-5.8) | 3.8 (2.2-6.4) |  | 18287.1 | 6 | 3.3 | 2.0 (0.8-4.9) | 2.9 (1.0-8.4) |
|  |  |  |  |  |  |  |  |  |  |  |  |

^a^ Adjusted for age, body mass index, white blood cell count, hemoglobin, alcohol intake, and bilirubin.

Abbreviation: PY, person year; SD, standard deviation.

^b^ Adjusted for confounders except bilirubin levels
