# Supplementary material for: Combined Effects of Smoking and Bilirubin Levels on the Risk of Lung Cancer in Korea: The Severance Cohort Study
Source: PLoS One. 2014 Aug 6;9(8):e103972. doi: 10.1371/journal.pone.0103972 (PMC4123988; doi:10.1371/journal.pone.0103972)
Supplement: Table S2 — HR and 95% CI for lung cancer according to smoking status and smoking amounts. (DOCX) [file pone.0103972.s004.docx]

Table S2. HR and 95% CI for lung cancer according to smoking status and smoking amount ^a^

|  | **Men** | | | | |  | **Women** | | | | |
| --- | --- | --- | --- | --- | --- | --- | --- | --- | --- | --- | --- |
|  | **PY** | **Lung** | **Rate per** | **HR (95% CI) ^b^** | **HR (95% CI)** |  | **PY** | **Lung** | **Rate per** | **HR (95% CI) ^b^** | **HR (95% CI)** |
|  |  | **cancer** | **10,000** |  |  |  |  | **cancer** | **10,000** |  |  |
|  |  |  |  |  |  |  |  |  |  |  |  |
| **Smoking status** |  |  |  |  |  |  |  |  |  |  |  |
| Never-smoker | 65107.1 | 17 | 2.6 | 1.0 | 1.0 |  | 270000.3 | 49 | 1.8 | 1.0 | 1.0 |
| Former smoker | 88484.8 | 48 | 5.4 | 1.6 (1.0-2.6) | 1.6 (1.0-2.6) |  | 8031.3 | 4 | 5.0 | 3.0 (1.1-8.5) | 3.0 (1.1-8.5) |
| Current smoker ^c^ |  |  |  |  |  |  |  |  |  |  |  |
| light/medium smokers | 63317.1 | 27 | 4.3 | 2.0 (1.1-3.6) | 2.2 (1.2-3.9) |  | 12801.1 | 4 | 3.1 | 2.2 (0.8-6.2) | 2.2 (0.8-6.2) |
| (<20 cigarettes/day) |  |  |  |  |  |  |  |  |  |  |  |
| heavy smokers | 97211.9 | 83 | 8.5 | 3.6 (2.2-5.9) | 4.0 (2.4-6.5) |  | 3407.1 | 2 | 5.9 | 3.0 (0.7-12.5) | 2.9 (0.7-12.4) |
| (≥20 cigarettes/day) |  |  |  |  |  |  |  |  |  |  |  |
|  |  |  |  |  |  |  |  |  |  |  |  |

^a^ Adjusted for age, body mass index, white blood cell count, hemoglobin, and alcohol intake.

Abbreviation: PY, person year; SD, standard deviation.

^b^ Additionally adjusted for bilirubin levels.

^c^ 942 subjects (men: 706 and women: 236) missing on smoking amount among current smoking were excluded.
